# Editorial Expression of Concern to: TGF-*β*1 exposure induces epithelial to mesenchymal transition both in CSCs and non-CSCs of the A549 cell line, leading to an increase of migration ability in the CD133^+^ A549 cell fraction

**DOI:** 10.1038/s41419-025-07780-0

**Published:** 2025-07-02

**Authors:** V. Tirino, R. Camerlingo, K. Bifulco, E. Irollo, R. Montella, F. Paino, G. Sessa, M. V. Carriero, N. Normanno, G. Rocco, G. Pirozzi

**Affiliations:** 1 Department of Experimental Medicine, Second University of Naples, Naples, Italy; 2 Department of Experimental Oncology, National Cancer Institute, Naples, Italy; 3 Department of Thoracic Surgery and Oncology, National Cancer Institute, Naples, Italy

Editorial Expression of Concern to: *Cell Death and Disease* 10.1038/cddis.2013.144, published online 02 May 2013

The Editors-in-Chief would like to alert the readers that concerns have been raised regarding two pairs of overlapping images in Fig. 2 (Untreated SP- and SP+; Treated CD133- and CD133+).

The authors have stated that the errors occurred during figure preparation and provided new data to validate the results presented in the article, which demonstrate similar results. However, as the original data are no longer available, readers are advised to interpret these results with caution.

V Tirino agrees to the aforementioned concerns, but not the wording of this Editorial Expression of Concern. None of the other authors have responded to any correspondence from the publisher about this Editorial Expression of Concern.

